# Regulated and Emerging Mycotoxins in Bulk Raw Milk: What Is the Human Risk?

**DOI:** 10.3390/toxins15100605

**Published:** 2023-10-09

**Authors:** Marta Leite, Andreia Freitas, Jorge Barbosa, Fernando Ramos

**Affiliations:** 1 Faculty of Pharmacy, Health Science Campus, University of Coimbra, Azinhaga de Santa Comba, 3000-548 Coimbra, Portugal; marta.leite@iniav.pt; 2 National Institute for Agricultural and Veterinary Research (INIAV), Rua dos Lágidos, Lugar da Madalena, 4485-655 Vila do Conde, Portugal; andreia.freitas@iniav.pt; 3 REQUIMTE/LAQV, R. D. Manuel II, Apartado, 4051-401 Oporto, Portugal

**Keywords:** raw milk, regulated mycotoxins, emerging mycotoxins, occurrence, exposure assessment

## Abstract

Mycotoxins are abiotic hazards whose contamination occurs at the pre- and post-harvest stages of the maize value chain, with animal exposure through contaminated feed leading to their excretion into milk. Currently, only aflatoxin M1 is regulated in milk products. Since feed materials and complete feed present a multi-mycotoxin composition and are the main mycotoxin source into milk, it is important to recognize the occurrence of multiple toxins and their co-occurrence in this highly consumed food product. The aim of this study was to determine the content of regulated and emerging mycotoxins in milk samples, which allowed for evaluating the occurrence and co-occurrence patterns of different mycotoxins known to contaminate feed materials and complete animal feed. Human exposure considering the occurrence patterns obtained was also estimated. Aflatoxins, fumonisins, zearalenone, and emerging mycotoxins were among the mycotoxins found to be present in the 100 samples analyzed. Concentrations ranged from 0.006 to 16.3 μg L^−1^, with no sample exceeding the AFM1 maximum level. Though several mycotoxins were detected, no exceeding values were observed considering the TDI or PMTDI. It can be concluded that the observed exposure does not pose a health risk to milk consumers, though it is important to recognize vulnerable age groups.

## 1. Introduction

Milk represents one of the most important components of a healthy and balanced human diet, being a highly consumed food product in all age groups [1,2]. It represents a source of animal protein and essential nutrients, especially for children [3]. Cattle milk, specifically, is the main type of milk used in the human diet, representing 81% of world production, followed by buffaloes with 15%, goats with 2%, sheep with 1%, and camels with 0.5% [4]. Due to its recognizable health and nutritional benefits, consumption of this dairy product without any processing, heating, or pasteurization is becoming increasingly popular in the European Union (EU) [5]. This subject is still a controversial subject, with official control entities recommending not consuming it in its raw form, since the risk of contamination is greater due to the higher potential presence of pathogens [5,6]. According to EU hygiene plans, Member States can restrict or prohibit the sale of raw milk for human consumption, though an increase in its consumption has been observed, with some EU countries allowing its sale through vending machines [7]. In some countries, such as France, Italy, and Greece, it is very common and, in some cases, promoted by governmental agencies. French supermarkets and grocery stores have this product available for sale, as also happens in Italy where its consumption has been part of their traditional culture. In Greece, dairy farmers are allowed to sell it directly to consumers, though it is not widely available in supermarkets. On the other hand, in countries such as Germany and the United Kingdom, this consumption is restricted due to safety reasons, but this scenario is gradually changing. Overall, the consumption of raw milk in European countries has been increasing, and it is becoming more accessible in many countries [6,8]. However, its safety is still a concern, and it is important to first recognize the health risk–benefit balance [6,9].

The quality and safety of this important dairy product is highly dependent on the animal diet, which is characterized by a very complex mixture of individual feed components, consisting of forages, concentrates, and preserved feeds. Different contaminations in each raw feed component can comprise a source of multi-mycotoxin exposure in the final animal diet, with maize silage and concentrated feeds representing the factors most correlated with mycotoxin contamination in milk [10,11]. For instance, maize silage represents 50 to 75% of the total daily intake (TDI) of feedstuffs in dairy farms [12,13]. These raw feed materials are prone to be contaminated mainly by *Fusarium* toxins, including deoxynivalenol (DON), zearalenone (ZEA), fumonisins (FBs), enniatins (ENNs), beauvericin (BEA), and nivalenol (NIV) [14,15]. Nonetheless, post-harvest contamination by *Penicillium* toxins such as mycophenolic acid (MPA), marcfortine A (MAC A), roquefortine C (ROQ C), and andrastatin A (AND A) can also occur [12,16]. Contamination of feedstuffs by mycotoxins can lead to economic losses and negative health impacts concerning animal husbandry [17]. Thus, the intake of contaminated feed can lead to the excretion of mycotoxins into milk, since once ingested by ruminants, these toxins are metabolized, biotransformed, and/or transferred to these animal products, becoming a risk to human health if consumed. Aflatoxin M1 (AFM1) is the most-studied mycotoxin in this food product, with an established maximum level of 0.05 µg kg^−1^ by the EU legislation [18]. It is also the only mycotoxin to which a maximum permitted level is established. Nonetheless, several studies have reported different mycotoxins in animal milk, which were linked to contaminated feed [19,20,21,22]. Ochratoxin A (OTA), fumonisin B1 (FB1), aflatoxins B1, B2, G1, and G2 (AFB1, AFB2, AFG1, and AFG2), and zearalenone (ZEA) comprise some examples of mycotoxic contaminants found in milk samples, though no maximum levels of these mycotoxins have been established [23]. Most of these fungal toxins are thermal resistant, with no effects on their occurrence when pasteurization, sterilization, or spray-drying are applied during processing, leading to their presence in the active form in processed milk and other dairy products, making it essential to evaluate and manage the mycotoxin levels either in the raw or processed dairy food to guarantee their quality and safety [24]. One of these examples are aflatoxins (AFs), which present a low decrease in concentration when submitted to pasteurization processes [25]. FB1 and FB2 have also demonstrated heat stability when pasteurization procedures (62 °C for 30 min) were applied to FB-contaminated milk samples [26].

The risk of emerging mycotoxins to humans is also an area of concern due to the potential health implications that they can have. Emerging mycotoxins are defined as toxins not routinely analyzed in laboratories with an increasing prevalence in several raw food materials and end-consumer products [27]. Generally, they are less studied than regulated mycotoxins, so there is a lack of data on their occurrence in food-based products, on the co-occurrence with other mycotoxins, and on their health effects in humans, which leads to a risk of exposure not fully understood [28]. Notwithstanding, their occurrence in raw feed materials and complete feed have already been reported at significant levels, with BEA and ENNs being frequent contaminants of livestock diets [29,30]. The European Food Safety Authority (EFSA) has also issued scientific opinions concerning the risks to human and animal health due to the presence of these mycotoxins in raw cereals, feedstuffs, and cereal-based foods, namely beauvericin (BEA), enniatins (ENNs), nivalenol (NIV), citrinin (CIT), and *Alternaria* toxins, such as tenuazonic acid (TEA) and tentoxin (TTX) [31,32,33,34].

In general, a broad range of mycotoxins occur in ruminant diets, with possible carry-over to milk, and the number of available studies concerning the occurrence and transfer of these fungi contaminants into this dairy product is still very scarce. Considering that multi-exposure to mycotoxins is the most likely scenario and could affect their toxicological effects in humans and animals, there is a need to determine the co-occurrence of mycotoxins. In this matter, the present work aimed at performing a first unique comprehensive assessment of regulated, non-regulated, and emerging mycotoxins in raw milk samples, through a previously validated analytical method via ultra-high-performance liquid chromatography coupled to tandem mass spectrometry (UHPLC-MS/MS) [35]. Occurrence analysis was performed to fully evaluate the mycotoxic content of this potential food product and co-occurrence studies were carried out to evaluate the complete pairing of full sampling (absolute frequency) and to assess the frequency of pairing in positive sampling (relative frequency). An exposure assessment of the identified mycotoxins was, finally, performed to measure the potential risk of the consumption, which will ultimately allow for establishing proper (co-)contamination patterns and produce the scientific basis for risk management strategies toward the mitigation of mycotoxin exposure.

## 2. Results and Discussion

### 2.1. Occurrence of Mycotoxins in Raw Bulk Milk

Raw bulk milk from Portuguese dairy farms was collected during the years of 2020 and 2021, and analyzed for the presence of 23 regulated, non-regulated, and emerging mycotoxins. The results from the mycotoxin occurrence in the 100 collected samples are shown in Table 1.

In total, 13 out of 23 analytes, at concentrations ranging from 0.006 to 16.3 µg L^−1^, were detected via UHPLC-MS/MS in raw cow milk. AFG1, AFG2, CIT, DON, MPA, NIV, OTA, PA, TEA, and TTX (*n* = 10) were not detected in any of the 100 samples, although previous studies have reported the contamination of raw milk by AFG1, AFG2, and OTA [21,36,37]. No studies concerning TEA and TTX toxins have been previously reported for milk samples, though they represent the main Alternaria mycotoxins reported in animal feed [31]. The mycotoxins that were detected comprise those produced by the main mycotoxin-producing fungi species, namely *Aspergillus* spp. (Afs) and *Fusarium* spp. (BEA, ENNs, and ZEA), and, except for AFM1, an AFB1-biotransformation product resulting from animal metabolism, all detected analytes are known to contaminate animal feed [38,39,40,41].

Ninety-seven percent of the samples (>LOD) analyzed were positive for mycotoxic contamination, with only three samples not presenting any mycotoxin in their composition. In order of magnitude concerning their percentage occurrences (>LOD), the following decreasing rating was observed: BEA (97%) > ENNA (88%) > ENNB (76%) > HT-2 (52%) > AFB2 (49%) > AFM1 (46%) > FB2 (39%) > ZEA (29%) > T-2 (28%) > AFB1 (23%) > FB1 (10%) > PAT (6%) > MON (5%). Considering the frequency of positive samples above the LOQ values, FBs and PAT did not present any contamination percentage in the analyzed samples, and MON and ZEA displayed 1% of positives. Overall, the emerging mycotoxins BEA and ENNs were the analytes found most frequently in raw milk, at percentages ranging from 76 to 97% (>LOD). The highest mean concentration level was also from emerging mycotoxins, namely ENNA and ENNB, with values of 10.16 and 5.97 µg L^−1^, respectively. ZEA presented one positive sample with 9.8 µg L^−1^. The total mean mycotoxin content in the 100 samples was 14.3 µg L^−1^, with a maximum value of 28.1 µg L^−1^ in a sample contaminated with 5 mycotoxins.

#### 2.1.1. Aflatoxins

AFM1 was one of the most prevalent aflatoxins detected in the 100 milk samples analyzed in this study. The analytical results showed that AFM1 was detected in 46 samples (>LOD) from a total of 100 raw milk samples, at an average concentration of 0.016 µg L^−1^ corresponding to the quantifiable positive samples (13%). A maximum value of 0.021 µg L^−1^ was found in one sample, which represents less than half of the maximum level established in the EU regulations for AFM1 in milk (0.05 µg kg^−1^) [18]. Concentration levels of this mycotoxin in cow milk samples, with values above the EU maximum level, were reported in previous studies [1,42,43,44]. In a Portuguese study on marketed milk, a contamination rate of 27.5% (positive samples above the LOD) was found, with a mean value of 0.023 ± 0.024 µg L^−1^, and two samples above the legal maximum limit [45]. In another recent study regarding raw milk samples from the north of Portugal, no report on the presence of AFM1 or other aflatoxins (AFB1 and AFB2) was referenced [46].

AFB1, AFB2, AFG1, and AFG2 present regulated maximum concentration levels for different commodities, including raw feed materials (e.g., raw maize-based products) and complete feedstuffs. Nonetheless, no maximum levels are established in regulatory frameworks for these AFs [18]. In this study, AFB1 and AFB2 were detected in 23 and 49% (>LOD) of the samples. Considering positive samples above the LOQ, the average concentration values were 0.08 (AFB1) and 0.06 (AFB2) µg L^−1^ in the 19 and 47 samples, respectively. From the AFB1-contaminated feed, only 1–6% of its concentration in dairy feed represents the concentration of AFM1 in milk, thus making its carry-over in the primary form into this dairy food product possible [25]. Scaglioni et al. [47] detected both mycotoxins (AFB1 and AFM1) in pasteurized and UHT milk samples at a concentration range of 0.7 to 1.5 µg L^−1^, though no AFB1 was detected in the raw and concentrated milk samples. AFB2 was also reported in one sample at a concentration of 0.045 µg L^−1^ in Nigerian cow milk samples [42]. In the same study, AFB1 was also present at a percentage of 4% at a maximum contamination level of 0.010 µg L^−1^. Mao et al. [37] also reported an occurrence of 12.4% in positive samples for AFB1 in 250 raw milk samples, with a maximum value estimated at 0.023 µg L^−1^. The authors also reported the contamination of one sample with AFG1 at a concentration of 12 µg L^−1^. In the present study, no contamination was observed with AFG1 or AFG2.

The presence of these toxins, which used to be considered of minor importance in European countries, can be justified due to climate changes in the last few decades [1,24]. High temperatures and long droughts can lead to an increasing incidence of AFB1 in animal feed with a possible consequent occurrence of AFM1 in milk [1]. The risk of human hepatocellular carcinoma (HCC), a leading cause of cancer death worldwide, is considered to be putatively increased by human exposure to chronic low levels of these type of mycotoxins [48,49]. Specifically, AFM1 is the primary metabolite of AFB1, with both being classified as Group 1 human carcinogens [50]. AFM1 is less toxic than AFB1, with its occurrence in milk mainly due to feed contamination by the latter, but also due to the rumen microbial ecosystem [37]. Although the data obtained in this study present a low frequency and low concentration of these compounds in raw milk, continuous monitoring is crucial to perceive the potential risk to consumers of this unprocessed food product, especially in children, since this age group is more susceptible to the adverse effects of mycotoxins.

#### 2.1.2. Fumonisins

Fumonisins B1 and B2 were detected in 39 and 10% of the total samples, all below the corresponding LOQs. Contamination in food and feed by this group of mycotoxins has been rising, with some reports on high prevalence and/or concentration rates in maize-based food and animal feed [15,51,52,53,54,55,56]. Caloni et al. [57] demonstrated that FB1 is poorly metabolized in the rumen of animals, which present an apparent tolerance toward this mycotoxin. A few works have already addressed the occurrence rate of FBs in milk samples. A study by Coffey et al. [58], that comprised a quantitative Monte Carlo exposure assessment model for mycotoxins in milk, estimated a mean concentration of 0.36 µg kg^−1^ of FB1 in milk. Gazzotti et al. [59] identified 8 positive samples in a total of 10 for this toxin, in different types of milk, with concentration values up to 0.32, 0.38, 0.43, and 0.38 µg kg^−1^ for raw milk, fresh whole milk, high-quality milk, and organic milk, respectively. In the present work, a moderate number of samples were positive above the respective LOD (0.68 (FB1) and 0.40 (FB2) µg L^−1^), though not quantifiable regarding the method’s LOQ values. Nonetheless, the indicative percentage values reveal the need to keep monitoring Portuguese milk samples, especially in a changing climatic scenario, since FBs are highly toxic for humans, with a suspected risk concerning esophageal and liver cancers, neural tube defects, and cardiovascular problems in humans [59].

#### 2.1.3. Trichothecenes

Regarding type-B trichothecenes (TCTs), no detection was observed for deoxynivalenol (DON) and nivalenol (NIV) in any of the 100 samples. A quantitative Monte Carlo exposure assessment model estimated that a possible carry-over of DON would generate a mean concentration of 1 µg kg^−1^ [58]. Winkler et al. [60] also detected DON at concentrations up to 2.5 µg L^–1^, after a feeding trial with contaminated feed at 0.07, 2.62, and 5.24 µg L^–1^ with a calculated carry-over ranging from 0 to 0.0017. Even so, according to other studies on the carry-over of DON into milk after artificially contaminating feed, the unmetabolized mycotoxin did not appear even with high levels of DON-contaminated feed [61]. The detection of its metabolite DOM-1 is, on the other hand, observed in these cases. For example, Sørensen and Elbæk [62] found up to 0.0003 µg kg^−1^ of DOM-1 in 5 of the 20 milk samples analyzed, with no detection of DON. The controversy regarding the effective DON carry-over into milk and the rate of metabolization into DOM-1 need further studies, with larger occurrence works on this important food product. The necessity for a comprehensive assessment on both mycotoxins will therefore give new insights on the risk of DON-contaminated feed on milk safety concerning type-B TCTs.

T-2 toxin, considered by the European Food Safety Authority as one of the most dangerous contaminants for human health, was detected in 28% of the milk samples (>LOD) with concentration levels averaging 1.7 µg L^−1^. On the other hand, HT-2 toxin presented a higher absolute frequency rate with 52 positive samples above the LOD, with a lower mean concentration value of 0.5 µg L^−1^ (44 samples > LOQ). Maximum concentration levels of 6.4 and 1.9 µg L^−1^ were found for T-2 and HT-2, respectively, in the samples analyzed in this study. Based on the study of Coffey et al. [58], a carry-over concentration for T-2 was estimated at 0.0722 µg kg^−1^. Also, Yoshizawa et al. [63] observed a recovery of 0.2% in milk after the administration of 156.9 mg of T-2 to a 375 kg lactating Jersey cow. In their study, the transmission of T-2 toxin into milk was found to be in the form of the parent compound and HT-2 along with six metabolites. In a study concerning 31 raw milk samples, T-2 was detected in only 1 sample at a concentration of 1.53 μg L^−1^ [46]. The values found in regard to T-2 and HT-2 toxins in our work could be due to a high contamination of the feeding system.

#### 2.1.4. Zearalenone

Zearalenone (ZEA), a known feed-contaminating *Fusarium* mycotoxin, was found in 29% of the samples (>LOD). One sample was quantifiable at a concentration of 9.8 µg L^−1^. According to the EFSA, the carry-over of ZEA into milk is negligible for quantities of feed contamination at low levels [64]. Other studies have also shown no or very low carry-over of this mycotoxin into milk, with their metabolites α- and β-ZEA being the most encountered in this food product [3]. Nonetheless, a few reports have demonstrated high levels of ZEA in these matrices. A study from Ecuador on 209 raw milk samples observed an absolute frequency of 99.5% of positive samples for ZEA, with a mean range of 1.5 µg L^−1^, and a maximum level of 10.2 µg L^−1^ [65]. Huang et al. [36] also observed an occurrence of ZEA in Chinese raw milk, liquid milk, and milk powder samples of 23.3, 16.7, and 25%, respectively. Though the mean concentration values were very low, namely of approximately 0.015, 0.021, and 0.012 μg kg^−1^, respectively. According to a stochastic simulation model, the ZEA concentration in raw milk is estimated at 0.125 μg L^−1^, considering a maximum carry-over rate of 2% [11].

Contrasting AFs and FBs, ZEA is not characterized as a carcinogen, presenting effects at the estrogenic activity level [66]. Due to the low carry-over rates into milk, the safety of this product can be perceived as not significant for human health [13]. Nonetheless, its potential adverse effects on cattle health are of great concern for dairy producers, as its presence in milk can be indicative of this feed safety issue.

#### 2.1.5. Emerging & Non-Regulated Mycotoxins

BEA (97%), ENNA (88%), and ENNB (76%) presented the highest percentage of positive samples, though the highest mean concentrations were verified for ENNA and ENNB, which ranged from 9.87 to 16.32 and 4.36 to 10.6 μg L^−1^, respectively. In contrast, BEA presented lower concentration values with a mean of 0.30 μg L^−1^, and a maximum level of 0.81 μg L^−1^. Data regarding the toxicity of these emerging group of mycotoxins indicate human adverse effects on the gastrointestinal tract, immunity, and steroidogenesis, with BEA revealing the highest cytotoxicity [67]. Its prevalence in different feed and food origins has been reported, though the representativeness of samples is predominantly small [34]. Regarding milk samples, the studies are still scarce, with the majority focusing on infant formulas, milk-based foods for children, and human breast milk. Nonetheless, a recent study on raw milk samples was performed in northern Portuguese samples, where very similar occurrence rates to the values obtained in our study were reported [46]. ENNs and BEA were evaluated for the first time in this matrix, with reports of their occurrence in 21 and 28 samples, respectively, in a total of 30 samples. A concentration range from 0.09 to 2.96 μg L^−1^ was obtained for BEA, and 0.03 to 4.14 μg L^−1^ for ENNs (ENNA, ENNB, ENNA1, and ENNB1). A Polish study of 76 raw milk samples was also recently performed, with 20 and 41% of the samples contaminated with BEA and ENNB, with maximum concentration levels of 6.17 and 0.85 μg kg^−1^, respectively [68]. These emerging mycotoxins are the most commonly found in the literature, with reports on the high levels present in feedstuffs and complete feed, though toxicology data are still scarce to be able to evaluate their effects [23].

The small lactone mycotoxins moniliformin (MON) and patulin (PAT) were also positive for 5 and 6% (>LOD), respectively. Only one sample contaminated with MON was above the LOQ value, presenting a concentration of 1.3 μg L^−1^, with no quantifiable samples for PAT. The occurrence of MON is worldwide, and it is present in maize with concentrations that can reach 20 mg kg^−1^ [69]. Its toxicology is not yet well understood but it has been characterized by provoking myocardiac damage [69]. On the other hand, PAT is a regulated mycotoxin but has only been associated with fruit juices, apple-based products, and baby foods other than processed cereal-based foods for infants and young children [18]. Exposure to PAT is associated with adverse neurological, immunological, carcinogenic, teratogenic, genotoxic, and gastrointestinal effects [70]. Nonetheless, this fruit-associated mycotoxin presents a concentration reduction when processing techniques, such as heating, radiation, or high pressure, are applied [71]. This is the first time MON and PAT were evaluated and reported in raw milk samples.

### 2.2. Co-Occurrence of Mycotoxins in Raw Bulk Milk

**Figure 1:**
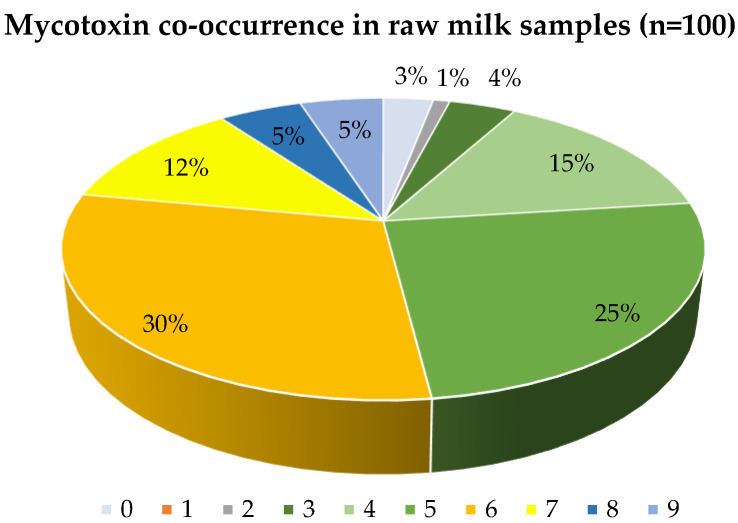
Mycotoxin co-occurrence in 100 Portuguese raw milk samples.

Co-contamination by multiple mycotoxins was found to be highly prevalent in Portuguese raw milk samples. The results concerning the prevalence data revealed the presence of two or more mycotoxins in 97% of the samples, with a high prevalence of emerging mycotoxins (Figure 1). Only three samples presented results lower than the limit of detection, being considered as non-contaminated by the group of mycotoxins analyzed in this study. Contamination with only one mycotoxin was not observed for none of the samples, and the maximum number of contaminants was nine. In total, 5% of the samples were contaminated with nine mycotoxins, and another 5% with eight mycotoxins. Previous studies revealed the co-occurrence of mycotoxins in raw animal milk from different sources in China, Spain, and Nigeria [36,37,42,72]. For example, Huang et al. [36] analyzed four mycotoxins (AFM1, OTA, ZEA, and α- zearalenol (α-ZOL)) in raw milk, liquid milk, and milk powder samples from dairy farms and supermarkets, and found multi-contamination by those mycotoxins at the percentages of 15, 45, and 22% for two, three, and four co-occurring mycotoxins.

**Figure 2:**
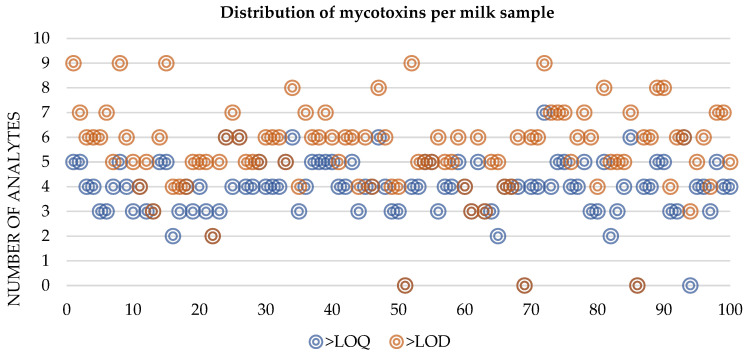
Distribution profile of the number of mycotoxins in the global sampling scheme.

The global distribution of co-occurring mycotoxins was observed between four (15%) and six (30%) mycotoxins in the same sample (Figure 2). The higher percentage (30%) corresponds to six mycotoxins co-occurring in the same samples, followed by 25% with five mycotoxins. Distribution following the LOQ values reveals the majority of co-occurring rates are between three and five mycotoxins in the same sample, with only 3% with six analytes and 1% with seven analytes. The average number of individual mycotoxic contaminants per sample was 5.6 (>LOD).

**Figure 3:**
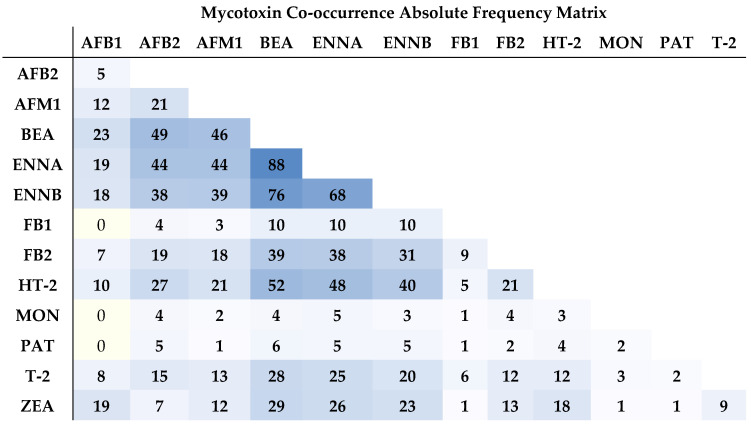
Co-occurrence matrix concerning mycotoxin pairing in 100 raw milk samples, expressed as percentage of absolute frequency (AF) (number of samples).

Among the 75 bi-combinations found, the most prevalent ones are represented by BEA + ENNA (88%), BEA + ENNB (76%), ENNA + ENNB (68%), and BEA + HT-2 (52%) (Figure 3). None of the samples presented AFB1 combined with FB1, MON, or PAT. Fumonisins B1 and B2 co-occurred in 9 of the samples, considering only a total of 10 positive samples for FB1. HT-2 and T-2 were also co-present in 12%, which is a low value taking into account the lowest mycotoxin positives of 28% (T-2). Nonetheless, as previously discussed, HT-2 is a metabolite of T-2, which leads to co-contamination with both mycotoxins depending on the metabolic rate of T-2, with both the metabolite and parent compound being excreted into milk after the ingestion of contaminated feed by these type-A TCTs.

**Figure 4:**
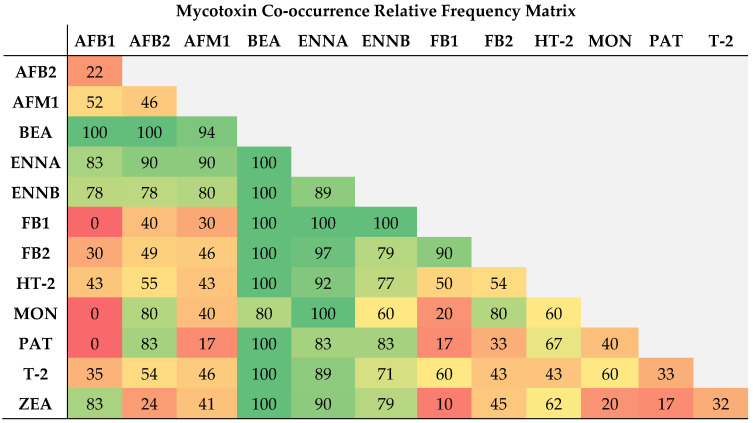
Co-occurrence frequency matrix concerning mycotoxin pairing in positive raw milk samples, expressed as percentage of the ratio of absolute frequency (AF) and total number of positive samples (mycotoxin lowest RF value).

To the authors’ knowledge, no studies regarding the co-occurrence of regulated and emerging mycotoxins in milk have been published so far. Notwithstanding, several co-occurrence studies on the main contributors for mycotoxin content in milk, namely maize silage and complete feed, have been reported. For instance, ZEA and DON occur as pre-harvest and post-harvest mycotoxins, often co-occurring in feed, and, therefore, with special relevance in animal nutrition [56,73]. Though no occurrence of DON was observed in any of the analyzed samples in the present study. In another study by Zachariasova et al. [74] concerning multiple mycotoxins in European feedstuffs, co-occurrence in the complex compound feed for dairy cows was observed for ZEA + FB1 at a percentage rate higher than 80%, which was also observed for feeding maize and maize silage components, though at lower rates (<20%). In our study, only 1% of the samples presented this mycotoxic pattern, which corresponds to 10% of the positive samples (Figure 4). Also, DON was the main co-occurring mycotoxin with the highest rates, though in the present study, no contamination was observed with this type-B TCT, as previously stated. According to Grenier and Oswald [75], synergistic or additive effects due to the co-presence of mycotoxins arise with AFs occurring with FBs, TCTs, OTA, and CIT. Frequencies of 7, 19, and 18% were found between AFB1 + FB2, AFB2 + FB2, and AFM1 + FB2, respectively. Lower co-occurring frequencies were found with FB1, namely 4% with AFB2 and 3% with AFM1. Higher global frequency values were found with AFs and type-A TCTs, namely HT-2 + AFB2 (27%) and HT-2 + AFM1 (21%). T-2 toxin was also found to co-occur with the detected AFs, at frequencies of 8 (AFB1), 15 (AFB2), and 13% (AFM1), respectively. In a wider study concerning the co-occurrence of regulated, masked, and emerging mycotoxins in finished feed and maize, a high frequency with regulated toxins (ZEA, DON, AFs) and emerging mycotoxins, namely ENNs, BEA, and MON [29] was observed. The authors observed correlations between ENNs and ZEA, while BEA with AFs, HT-2, and T-2 toxins. MON was also found to be positively correlated with FBs, DON, and ZEA. In our study, from 29 positive samples for ZEA, 26 (90%) and 23 (79%) were also positive for ENNA and ENNB, respectively. Taking into consideration the positive samples, ENNs also present high co-occurrences with FB1 at a frequency of 100%. ENNs and AFs were equally found in a co-occurrence frequency range between 78 and 90%. The most prevalent pairings are represented by the emerging mycotoxin BEA, which presents 100% of co-occurrence with all mycotoxins, except with MON (80%) and AFM1 (94%).

### 2.3. Assessment of Human Exposure to Mycotoxins

The estimated daily intake (EDI) was based on a deterministic approach for the determination of human exposure to mycotoxins detected as occurring in milk samples. The EDI was estimated via three different methods, namely taking into account only the positive samples and the corresponding mean and maximum values, and by using the most common approaches in mycotoxin studies, which consider non-detected samples by using upper bound (UB) and lower bound (LB) approaches. According to the European Food Safety Authority (EFSA), when using the LB approach, samples below the LOD are replaced by zero and samples below the LOQ by the corresponding LOD value; when using the UB approach, samples below the LOD and LOQ are designated with the corresponding LOD and LOQ values, respectively [76]. The EDIs obtained concerning the intake of mycotoxins by the general population are presented in Table 2, and were evaluated taking into consideration an average human body weight of 79.5 kg and a human consumption of milk per capita in the year of 2021 of 66 kg per habitant per year (average of 0.18 kg per habitant per day), as according to the database published by the Portuguese National Statistics Institute (INE) [77].

The estimated intake of AFM1 through milk consumption of an average Portuguese adult citizen was estimated at 0.01 ng kg^−1^ bw day^−1^ (LB), and, in the worst-case scenario, at a concentration of 0.03 ng kg^−1^ bw day^−1^ (UB). At the European level, the Joint FAO/WHO Expert Committee on Food Additives (JECFA) evaluated the daily intake of this widely studied mycotoxin through milk consumption concerning regional diets, estimating an EDI of 0.11 ng kg^−1^ bw day^−1^ for the European diet, which is ten times higher than the EDI (LB) obtained in this study [78]. Portuguese data on the exposure assessment of AFM1 due to the consumption of milk have also been previously studied in pasteurized and UHT samples [45]. In this work, a higher estimated intake was observed for the general Portuguese population, namely at 0.08 ng kg^−1^ bw day^−1^. Regarding raw milk samples, other European countries have shown similar values of average daily intake to the ones found in our study, namely in ranges of 0.0122–0.0568 ng kg^−1^ bw day^−1^ (Croatia), 0.0063–0.0243 ng kg^−1^ bw day^−1^ (France), and 0.0135–0.0538 ng kg^−1^ bw day^−1^ (Italy, considering UHT, pasteurized, raw, and conventional and organic milk) [25]. Considering a mix of different types of milk, including UHT, pasteurized, raw cow and/or conventional, organic, and kid’s milk, Greece and Serbia presented higher values of daily intake, namely of 0.1838 and 0.6891 ng kg^−1^ bw day^−1^, respectively [25].

The highest exposure observed for regulated mycotoxins was from ZEA, with an EDI of 1.71 and 9.51 ng kg^−1^ bw day^−1^, though it is important to disclose that only one sample was positive for this mycotoxin above the LOQ. In addition to the AFM1 mycotoxin, AFB1 and AFB2 presented the lowest exposure estimation at values of 0.03 (LB) and 0.05 (UB) ng kg^−1^ bw day^−1^, and of 0.06 ng kg^−1^ bw day^−1^ (LB and UB), respectively. Emerging mycotoxins ENNA and ENNB presented the highest exposure concentration, ranging from 10.28 (ENNB/LB) to 22.83 (ENNA/UB) ng kg^−1^ bw day^−1^. The values of ENNA were approximately twice the EDIs for ENNB, either for the LB or UB approach. Overall, a range of 0.01 to 20.34 ng kg^−1^ bw day^−1^ for the LB approach, and of 0.05 to 22.83 ng kg^−1^ bw day^−1^ for the UB approach, were obtained as the first-time estimation of multi-mycotoxin exposure in milk samples.

### 2.4. Risk Characterization

A risk characterization was, finally, performed, resulting from the previous data regarding mycotoxin exposure. In this matter, the outputs of exposure, namely the EDI values, were compared with the reference dose values. A hazard quotient (HQ) was therefore calculated on the basis of these data and the TDI and PMTDI values, when available, as established by the Joint Expert Committee on Food Additives (JECFA) of the FAO and WHO [79]. The TDI and PMTDI values are listed in Tables S1 and S2, as well as the calculated HQ for both the total number of positive samples and the total number of samples. HQ values above 1 (100%) indicate a health risk due to the levels of mycotoxin exposure concerning milk consumption by the target population; HQ values lower than 1 (100%) indicate the individual or target population are unlikely to suffer a health risk from the exposure levels to a certain mycotoxin due to milk consumption [50].

Concerning the HQ for AFs, it was not possible to compare the exposure estimates obtained in this work to national or European accepted metrics, since no TDI has been set for AFM1 or any other AFs by an international standard-setting institution which is explained due to the high toxicity of this group of mycotoxins [58]. In this matter, it is not possible to verify if the exposures to AFs can cause negative effects on human health, though in vivo and in vitro data suggest induced adverse effects by AFM1. Therefore, exposure to these mycotoxins should be minimized, adding to possible co-exposure leading to synergistic effects [80]. TDI values for emerging mycotoxins (ENNs, MON, CIT) have also not been established. Nonetheless, the JECFA has set the TDI and/or PMTDIs for DON, FB1 and FB2 (sum or single toxin), and T-2 and HT-2 toxins (sum or single toxin), which allowed for the calculation of the HQ for these mycotoxins.

The HQ values obtained ranged from 0.03 (sum of FB1 and FB2) to 19% (sum of T-2 and HT-2 toxins). Individually, ZEA presented the highest HQ, though 25 times lower than the threshold for a putative risk. Generally speaking, none of the exposure levels found exceed the TDI or the PMTDI values (all HQ below 1), indicating no associated health risk for the intake of these mycotoxins through milk consumption, in each of the approaches.

## 3. Conclusions

This study highlighted the importance of assessing the multi-mycotoxin content and subsequent exposure due to raw milk consumption. To the authors’ knowledge, this is the first time a co-occurrence study and exposure assessment was performed for regulated and emerging mycotoxins in raw milk. A low risk of mycotoxin from this important food product was found, though it is important to recognize co-contamination patterns that can lead to synergistic or additive effects. The most commonly found mycotoxins in raw milk included the regulated AFs, ZEA, HT-2, and T-2 toxins, with a high occurrence mainly due to emerging mycotoxins (BEA and ENNs). AFM1, the mycotoxin with the most well-documented threat to human health, was not present in any samples above half the regulatory limit. The presence of these toxins in milk, mainly due to animal exposure to contaminated feed, reveals the importance of implementing strategies and measures to reduce the risk of mycotoxin contamination in dairy farms, namely by developing better agricultural and farming management practices (e.g., sanitation of milking environment), testing feed for mycotoxic contamination, and applying proper storage and handling techniques. At a consumer level, the insurance of the raw milk source is also an added prevention strategy for its consumption, namely by applying heating procedures before its consumption.

## 4. Materials and Methods

### 4.1. Chemicals and Reagents

Reagents of analytical grade were mainly used, with the exception of mobile phase reagents which were of high-performance liquid chromatography (HPLC) grade. Analytical standards comprised: MPA and NIV from Supelco (Bellefonte, PA, USA); PA from Santa Cruz Biotechnology (Dallas, TX, USA); AFB1, B2, G1, G2, and M1, BEA, CIT, DON, ENNA, and B, FB1 and B2, HT-2 and T-2 toxins, MON, OTA, PAT, TEA, TTX, and ZEA from Sigma-Aldrich (Steinheim, Germany). Ultrapure H_2_O was supplied from Millipore System (Paris, France), C18 sorbent from Agilent Technologies (Santa Clara, CA, USA), acetonitrile (ACN) from Carlo Erba (Val de Reuil Cedex, France), anhydrous magnesium sulfate (MgSO_4_) and sodium chloride (NaCl) from Honeywell (Seelze, Germany), and formic acid from Chem-Lab (Zedelgem, Belgium). ACQUITY UPLC^®^ HSS T3 1.8 μm (2.1 × 100 mm i.d.) was purchased from Waters (Milford, MA, USA), and HPLC vials and Syringeless Device Mini UniPrep filters (0.45 μm PVDF, polypropylene) from Whatman (Maidstone, UK).

### 4.2. Sampling

A total of 100 raw cow milk samples were collected from the main Portuguese dairy region in the north of Portugal, corresponding to 100 dairy farms, in the years of 2020 and 2021. Sampling was performed directly from bulk milk cooling tanks (approximately 1 L per sample), in sterile labeled screwed-top bottles, in a 1 L volume. Each sample was gently homogenized and divided into batches for storage at −20 ± 2 °C, until further analysis. Subsamples (4 mL) were submitted for the analysis of regulated and emerging mycotoxins (*n* = 23) via a validated QuEChERS (Quick Easy Cheap Effective Rugged Safe) procedure. For identification and quantification purposes, an ultra-high-performance liquid chromatography triple quadrupole-linear ion trap coupled to a mass spectrometry system (UHPLC-QTRAP-MS/MS) was used.

### 4.3. Mycotoxin Extraction

Extraction of regulated and emerging mycotoxins was based on the previously reported protocol on the validation of these compounds in raw milk [35]. Briefly, a volume of 4.0 ± 0.1 mL of each raw milk sample was mixed with 16 mL of ACN:H_2_O (80:20, *v*/*v*), and submitted to automatic homogenization through a horizontal shaker for 60 min. After adding 0.5 g of NaCl and 2.0 g of MgSO_4_ (1:4, *w*/*w*) for partitioning purposes, the mixture was stirred for 1 min and centrifuged for 10 min, at 4500× *g*, and at a temperature of 4 ± 0.1 °C. An amount of 10 mL of the upper layer was mixed with 150 mg of C18 sorbent and 900 mg of MgSO_4_ for dispersive solid-phase extraction (dSPE), and further centrifuged at the same conditions as previously described. The supernatant was collected and completely dried under a nitrogen stream by using a Turbovap Zymark Evaporator system from Biotage (Hopkinton, MA, USA), and finally, reconstituted with 40% ACN in a final volume of 500 µL. The extract was transferred to HPLC filter vials for injection in the UHPLC-MS/MS system.

### 4.4. Mycotoxin Analysis

Mycotoxin analysis of raw cow milk samples was also performed as previously described in [35]. Succinctly, chromatographic separation was performed by using an ACQUITY UPLC^®^ HSS T3 1.8 µm (2.1 × 100 mm i.d.) column on a UHPLC Nexera X2 Shimadzu system (Shimadzu, Kyoto, Japan). Column oven was operated at 30 °C and the autosampler at 10 °C, with an injection volume of 20 µL at a flow rate of 0.2 mL min^−1^. Mobile-phase composition and gradient elution program were as follows: (A) 0.1% formic acid and (B) acetonitrile; gradient elution protocol, 95% A to 30% A (15 min), 30% A to 0% A (5 min, 2 min hold), 0% A to 95% A (3 min); run time: 25 min. For mass spectrometry analysis, Triple QTRAP 5500+ detector (Sciex, Foster City, CA, USA) was used, with the sequential mass detector (UHPLC-MS/MS) operated in a single run in positive and negative ion mode in a single run (ESI^+^/ESI^−^) through an electrospray interface (Turbo Ion Spray). Multiple reaction monitoring (MRM) parameters and ion transitions were previously optimized and defined for each compound [81]. For data acquisition and evaluation, the following software was used: Analyst^®^ and MultiQuantTM (Sciex, Foster City, CA, USA). Samples with results lower than the LOD values were considered negative for contamination, and samples with values ranging between the LOD and LOQ were attributed with the corresponding LOQ values.

### 4.5. Descriptive & Statistical Analysis

Descriptive data analysis was performed to calculate the absolute and relative frequencies of contaminated samples per total of analyzed samples. Co-occurrence studies were evaluated by absolute frequency in percentage considering the total number of samples (contaminated and non-contaminated samples), and by relative frequency considering the total number of positive samples (only contaminated > LOD values). Microsoft Excel^®^ was used to fulfil the purpose.

### 4.6. Estimated Daily Intake (EDI) & Hazard Quotient (HQ)

The daily intake of contaminants is dependent on the contaminant concentration in food and the daily consumption of the corresponding food in analysis. Human exposure assessment was therefore determined for the mycotoxins detected in the analyzed samples by calculating the estimated daily intake (EDI), which was based on the following equation:

EDI (µg kg^−1^ bw/day) = (C_toxin_ × D)/BW (1)

where C_toxin_ represents the mean concentration of the mycotoxin in milk (µg kg^−1^), D refers to the daily human consumption of milk (µg milk per day), and BW refers to the average individual body weight (kg).

EDI was based on a deterministic method that combined the average body weight for adult population (considered as 79.5 kg for the Portuguese population), the daily consumption of milk (0.18 kg per Portuguese inhabitant per day), and mycotoxin’s concentration level (ng g^−1^) at the lower-, average-, and upper-case scenarios.

Risk characterization was performed by calculating hazard quotient (HQ) as according to the International Agency for Research on Cancer (IARC) [51], based on the following equation:

HQ = EDI/(TDI or PMTDI)(2)

where TDI is the tolerable daily intake, which represents the amount of a chemical that can be ingested daily by humans without or at a low risk, allowing for evaluating the appropriate safe exposure levels, and PMTDI is the provisional maximum tolerable daily intake. If HQ is equal or higher than 1, i.e., EDI exceeds TDI or PMTDI values, it is considered that there is a risk of exposure to a certain contaminant; if HQ is lower than 1, i.e., EDI does not exceed TDI or PMTDI values, it is considered that there is no risk of exposure.

## Figures and Tables

**Table 1:** Mycotoxin data in raw milk samples from Portugal (*n* = 100).

Mycotoxins	% Positive Samples (>LOD)	% Positive Samples (>LOQ)	Maximum Value (µg L^−1^)	Range (µg L^−1^)	Mean Concentration of Positive Samples (µg L^−1^)
AFB1	23	19	0.20	0.018–0.20	0.08
AFB2	49	47	0.23	0.006–0.23	0.06
AFM1	46	13	0.02	0.011–0.02	0.02
BEA	97	92	0.81	0.09–0.81	0.30
ENNA	88	88	16.32	9.78–16.32	10.16
ENNB	76	75	10.60	4.36–10.60	5.97
FB1	10	0	-	-	-
FB2	39	0	-	-	-
HT-2	52	44	1.91	0.086–1.91	0.50
MON	5	1	1.30	-	-
PAT	6	0	-	-	-
T-2	28	11	6.39	0.90–6.39	1.70
ZEA	29	1	9.80	-	-

**%**—Percentage; AFB1—Aflatoxin B1; AFB2—Aflatoxin B2; AFM1—Aflatoxin M1; BEA—Beauvericin; ENNA—Enniatin A; ENNB—Enniatin B; FB1—Fumonisin B1; FB2—Fumonisin B2; MON—Moniliformin; LOD—Limit of Detection; LOQ—Limit of Quantification; PAT—Patulin; ZEA—Zearalenone.

**Table 2:** Estimated daily intake (EDI) for consumption of mycotoxins in milk, expressed in ng kg^−1^ bw day^−1^.

	Total Number of Positive Samples (ng kg^−1^ bw Day^−1^)		Total Number of Samples (ng kg^−1^ bw Day^−1^)	
Mycotoxins	EDI (Mean Level)	EDI (Maximum Level)	EDI (LB)	EDI (UB)
AFB1	0.18	0.45	0.03	0.05
AFB2	0.13	0.53	0.06	0.06
AFM1	0.04	0.05	0.01	0.03
BEA	0.69	1.84	0.64	0.65
ENNA	23.12	37.11	20.34	22.83
ENNB	13.58	24.13	10.28	12.59
FB1	**	**	0.15	2.09
FB2	**	**	0.35	5.43
HT-2	1.16	4.34	0.51	0.53
MON	*	*	0.09	1.57
PAT	**	**	0.08	2.41
T-2	3.9	14.5	0.54	1.27
ZEA	*	*	1.71	9.51
Sum of T-2 and HT-2	5.02	18.87	1.06	1.80
Sum of FB1 and FB2	-	-	0.50	7.52

* Only one positive sample above LOQ. ** No positive samples above LOQ values.

## Data Availability

Not applicable.

## Supplementary Materials

The following supporting information can be downloaded at: https://www.mdpi.com/article/10.3390/toxins15100605/s1, Table S1: hazard quotient values for mycotoxin consumption in milk samples, for total number of positives (mean and maximum concentrations) and for total number of samples (*n* = 100) (lower (LB) and upper bound (UB)) considering TDI; Table S2: hazard quotient values for mycotoxin consumption in milk samples, for total number of positives (mean and maximum concentrations) and for total number of samples (*n* = 100) (lower (LB) and upper bound (UB)) considering PMTDI.
