# Supplementary material for: Regulated and Emerging Mycotoxins in Bulk Raw Milk: What Is the Human Risk?
Source: Toxins (Basel). 2023 Oct 9;15(10):605. doi: 10.3390/toxins15100605 (PMC10610745; doi:10.3390/toxins15100605)
Supplement: Supplementary file 1 [file toxins-15-00605-s001.zip › toxins-2643846-supplementary.pdf]

toxins

Supplementary Materials: Regulated and Emerging Mycotoxins in Bulk Raw Milk: What Is the Human Risk?

Marta Leite, Andreia Freitas, Jorge Barbosa and Fernando Ramos

Table S1. Hazard Quotient values for mycotoxin consumption in milk samples, for total number of positives (mean and maximum concentrations) and for total number of samples (n=100) (lower (LB) and upper bound (UB)) considering TDI.

		Total number of positive samples		Total number of samples	
	TDI (ng kg^{-1} bw day^{-1})[1]	HQ (Mean)	HQ (Maximum)	HQ (LB)	HQ (UB)
**AFB1**	-	No TDI established			
**AFB2**	-	No TDI established			
**AFM1**	-	No TDI established			
**BEA**	-	No TDI established			
**ENNA**	-	No TDI established			
**ENNB**	-	No TDI established			
**FB1***	-	-	-	-	-
**FB2***	-	-	-	-	-
**HT-2****	-	-	-	-	-
**MON**	-	No TDI established			
**PAT**	400	-	-	0.0002 (0.02%)	0.006 (0.6%)
**T-2****	-	-	-	-	-
**ZEA**	250	-	-	0.007 (1%)	0.038 (4%)
**Sum of FB1 and FB2***	2000	-	-	0.0003 (0.03%)	0.004 (0.4%)
**Sum of T-2 and HT-2****	100	0.050 (5%)	0.189 (19%)	0.011 (1%)	0.018 (2%)

AFB1 – Aflatoxin B1; **AFB2** – Aflatoxin B2; **AFM1** – Aflatoxin M1; **BEA** – Beauvericin; **ENNA** – Enniatin A; **ENNB** – Enniatin B; **FB1** – Fumonisin B1; **FB2** – Fumonisin B2; **HQ** – Hazard Quotient; **LB** – Lower Bound; **MON** – Moniliformin; **PAT** – Patulin; **TDI** – Tolerable Daily Intake; **UB** – Upper Bound; **ZEA** – Zearalenone.

* HQ values are calculated as the sum of FB1 and FB2.

** HQ values are calculated as the sum of T-2 and HT-2.

[1] Joint FAO/WHO Expert Committee on Food Additives (JECFA) *Evaluation of Certain Mycotoxins in Food: Fifty-Sixth Report of the Joint FAO/WHO Expert Committee on Food Additives*; 2001.

Table S2. Hazard Quotient values for mycotoxin consumption in milk samples, for total number of positives (mean and maximum concentrations) and for total number of samples (n=100) (lower (LB) and upper bound (UB)) considering PMTDI.

		Total number of positive samples		Total number of samples	
	PMTDI (ng kg^{-1} bw day^{-1})[1]	HQ (Mean)	HQ (Maximum)	HQ (LB)	HQ (UB)
**AFB1**	**-**	No PMTDI established			
**AFB2**	**-**	No PMTDI established			
**AFM1**	**-**	No PMTDI established			
**BEA**	**-**	No PMTDI established			
**ENNA**	**-**	No PMTDI established			
**ENNB**	**-**	No PMTDI established			
**FB1***	**2000**	-	-	0.0001 (0.01%)	0.001 (0.1%)
**FB2***	**2000**	-	-	0.0002 (0.02%)	0.003 (0.3%)
**HT-2****	**60**	0.02 (2%)	0.072 (7%)	0.009 (0.9%)	0.01 (1%)
**MON**	**-**	No PMTDI established			
**PAT**	**400**	-	-	0.0002 (0.02%)	0.01 (1%)
**T-2****	**60**	0.06 (6%)	0.24 (24%)	0.009 (0.9%)	0.02 (2%)
**ZEA**	**500**	-	-	0.003 (0.3%)	0.02 (2%)
**Sum of FB1 and FB2***	**100**	0.05 (5%)	0.19 (19%)	0.011 81%)	0.02 (2%)
**Sum of T-2 and HT-2****	**-**	-	-	-	-

AFB1 – Aflatoxin B1; **AFB2** – Aflatoxin B2; **AFM1** – Aflatoxin M1; **BEA** – Beauvericin; **ENNA** – Enniatin A; **ENNB** – Enniatin B; **FB1** – Fumonisin B1; **FB2** – Fumonisin B2; **HQ** – Hazard Quotient; **LB** – Lower Bound; **MON** – Moniliformin; **PAT** – Patulin; **PMTDI** – Provisional Maximum Tolerable Daily Intake; **UB** – Upper Bound; **ZEA** – Zearalenone.

* HQ values are calculated as the sum of FB1 and FB2.

** HQ values are calculated as the sum of T-2 and HT-2.

[1] Joint FAO/WHO Expert Committee on Food Additives (JECFA) *Evaluation of Certain Mycotoxins in Food: Fifty-Sixth Report of the Joint FAO/WHO Expert Committee on Food Additives*; 2001.
